# Broad-spectrum resistance to fungal foliar diseases in wheat: recent efforts and achievements

**DOI:** 10.3389/fpls.2024.1516317

**Published:** 2024-12-13

**Authors:** Amira M. I. Mourad, Asmaa A. M. Ahmed, P. Stephen Baenziger, Andreas Börner, Ahmed Sallam

**Affiliations:** ^1^ Genebank Department, Leibniz Institute of Plant Genetics and Crop Plant Research (IPK), Seeland, Germany; ^2^ Department of Agronomy, Faculty of Agriculture, Assuit University, Assiut, Egypt; ^3^ Department of Genetics, Faculty of Agriculture, Assuit University, Assiut, Egypt; ^4^ Department of Agronomy and Horticulture, University of Nebraska–Lincoln, Lincoln, NE, United States

**Keywords:** wheat stripe rust, wheat leaf rust, wheat stem rust, wheat powdery mildew, gene enrichment, genome-wide association study, functional annotation

## Abstract

Wheat (*Triticum* spp.) is one of the most important cereal crops in the world. Several diseases affect wheat production and can cause 20-80% yield loss annually. Out of these diseases, stripe rust, also known as yellow rust (*Puccinia striiformis* f. sp. *tritici*), stem rust (*Puccinia graminis* f. sp. *tritici*), leaf rust (*Puccinia recondita*), and powdery mildew (*Blumeria graminis* f. sp. *tritici*) are the most important fungal diseases that infect the foliar part of the plant. Many efforts were made to improve wheat resistance to these diseases. Due to the continuous advancement in sequencing methods and genomic tools, genome-wide association study has become available worldwide. This analysis enabled wheat breeders to detect genomic regions controlling the resistance in specific countries. In this review, molecular markers significantly associated with the resistance of the mentioned foliar diseases in the last five years were reviewed. Common markers that control broad-spectrum resistance in different countries were identified. Furthermore, common genes controlling the resistance of more than one of these foliar diseases were identified. The importance of these genes, their functional annotation, and the potential for gene enrichment are discussed. This review will be valuable to wheat breeders in producing genotypes with broad-spectrum resistance by applying genomic selection for the target common markers and associated genes.

## 1 Introduction

Wheat (*Triticum* spp.) occupies an important place among other cereals crops worldwide in feeding the world. During its life cycle, wheat suffers from many diseases that infect its foliar parts. Different pathogens cause these foliar diseases which could be fungal, bacterial, or viral pathogens. Fungal diseases such as powdery mildew and different rusts are the most common foliar diseases in wheat. The main danger of these diseases is the ability to spread quickly and easily among plants once the infection occurs (Esmail et al., 2023a). Another important threat is that foliar diseases can also make wheat plants more vulnerable and very susceptible to other biotic and abiotic stresses such as insect infestations and drought stress (Figueroa et al., 2018). All of these problems lessen the productivity of grain yield and total production in wheat causing a significant reduction in all important component of yield. Climate change is expected to increase the pathogen spread (Kissoudis et al., 2014). Therefore, the chances of agriculture crops encountering infection with foliar disease singly or in combination are expected to be more frequent than in the past decades. This change will significantly deteriorate staple crops such as wheat, which is used as food and provides 20 percent of the calories and 20 percent of the protein in human diets.

Fungicide application can be used to control foliar diseases in wheat, but it can raise human health problems over long-term use. Furthermore, fungal pathogens have the ability to produce new races that overcome these fungicides. Therefore, there is a great interest in establishing approaches by which wheat genotypes resistant to foliar diseases singly or in combination can be developed through breeding programs (Mourad et al., 2018a; Abou-Zeid and Mourad, 2021; Bhavani et al., 2021; Abo-elyousr et al., 2022). Improving disease resistance will be more fruitful and cost effective than using fungicide application because it reduced input costs, does not rely on fungicides which may or may not be accessible to all growers, and is less harmful to the environment. Plant breeding can also produces cultivars that are likely to be resistant to broad spectrum against different races and species.

The resistance of foliar diseases is classified by two stages: seedling-plant resistance (also known as all-stage resistance “ASR”), and adult plant resistance “APR”. The main difference between these two classifications is that ASR produces a high level of resistance in all stages of plant development from seedling to adult plant resistance. Adult plant resistance is effective in a specific stage of plant development, namely the adult plant, and resistance usually begins at the boot stage (Roelfs et al., 1992). All-stage resistance is also known as “boom and bust cycles” due to the probability of the resistance gene when deployed singly being defeated by new pathogen races. Using a single gene for the resistance to these diseases in wheat was found generally not to be durable in agriculture due to the emergence of new virulent races (Ellis et al., 2014). To increase durability, gene pyramiding has been suggested as a highly effective strategy (Bajgain et al., 2015).

Identifying the key genes controlling foliar diseases can accelerate the genetic improvement of the resistance in wheat. Genome-wide association studies (GWAS) have played a key role in identifying new genes associated with different stem rust, stripe rust, leaf rust, and powdery mildew races in different parts of the world (Kang et al., 2019; Prasad et al., 2020; Alemu et al., 2021; Du et al., 2021; Hinterberger et al., 2022; Jin et al., 2022; Kaur et al., 2023). It is crucial to find genomic regions that show broad resistance to various races for the same diseases or multiple foliar diseases to reduce the breeding population sizes in cultivar development programs. In the last decade, many GWAS studies have reported important candidate gene models for the genes that resist different races of stem rust, leaf rust, and stripe rust, however, very few have been conducted to identify genes associated with different races in powdery mildew. Based on the previous resistance gene research, it is feasible to look for the common candidate genes to multiple diseases that have been reported for single foliar disease resistance in wheat. The identification of common genes that resist different races and pathogens across many countries will facilitate gene pyramiding with fewer genes, hence easier to breed cultivars that can fight the new emerging races due to the consequences of climate change.

Many previous reviews were done on specific diseases in wheat including the important resistance genes, mapping resistant QTL, and cloning of resistance genes. These reviews, however, focused on a specific disease (De Wit et al., 2009; Lo Presti et al., 2015; Figueroa et al., 2018; Prasad et al., 2020). Here, the present review sheds light on all research efforts that have been conducted in foliar disease resistance in wheat in the period between 2019 to 2023 focusing on the common genes and markers that were found to be significantly associated with different races of each pathogen as well as multiple pathogens in different parts of the world.

## 2 Recent advances in the phenotyping of foliar diseases

Precise and repeatable phenotyping is the key to a successful identification of the candidate genes associated with the target traits because it is directly related to the heritability of the trait. Disease resistance in wheat is normally scored using a simple visual scale extending from 0 (immune) to 9 (most susceptible). Furthermore, it was recommended that one person phenotypes the resistance to obtain consistent, repeatable, and accurate results because visual scoring relies on the precision of the human scorer (Sallam et al., 2015). Visual scoring is widely used in breeding for disease resistance and GWAS studies to identify candidate genes associated with disease resistance in wheat. These traditional phenotyping methods can be used to score a limited set of genotypes which also can be an obstacle in dissecting the genetic control of disease resistance by reducing the ability to identify minor and major genes affecting the disease resistance (Furbank and Tester, 2011).

The recent advances in high-throughput phenotyping (HTP) methods provide precise objective information on the interplay between hosts and their pathogens which sheds light on the development of durable resistance crop cultivars (Mutka and Bart, 2015). The advantages of HTP strategies are non-destructive tools and provide accurate high-dimensional and acquisition of large-scale phenotypic scores during each measured stage in plant development (Houle et al., 2010). Using such big and accurate data will be very useful for GWAS and QTL mapping method to identify promising candidate genes associated with target traits and explore the genetic basis of abiotic/biotic stress (Klukas et al., 2014).

In disease resistance, HTP has been successfully utilized to identify and quantify the symptoms of various foliar diseases. Multi-spectral and hyperspectral sensing, 3D scanning, fluorescence imaging, thermal and near-infrared sensing, and RGB imaging are examples of HTP that have been used to study plant disease resistance (Mahlein, 2016). In wheat, HTP was used to phenotype foliar disease resistance (**Table 1**). Few studies have utilized HTP for precious phenotyping of the foliar disease resistance in wheat in genome-wide association studies. This result may be due to the fact that most of the research breeders, especially those in developing countries, cannot afford the high cost of HTP equipment and data support.

**Table 1:** List of new phenotyping methods used in screening important foliar disease in wheat.

Disease	Screening method	Reference
Stripe rust	light-induced fluorescence spectroscopy	Atta et al. (2023)
	Remote Sensing	Wang et al. (2012)
	Deep Learning with Semi-Automated Image Labeling	Pretorius et al. (2017)
	proximal phenotyping and machine learning	Tang et al. (2023)
	Very high (spatial and temporal) resolution satellite (VHRS) and high-resolution unmanned aerial vehicle (UAV) imagery	Koc et al. (2022)
	Deep Residual Neural Networks	Hickey et al. (2012)
	Unmanned Aircraft Systems UAV remote sensing	Zhang et al. (2019a)
	UAV-Based Red-Green-Blue Imagery Ground and UAV Vegetation Indexes	Blasch et al. (2023)
	RGB and thermal sensors	Schirrmann et al. (2021)
Leaf rust	Hyperspectral Remote Sensing	Huang et al. (2007); Guo et al. (2021); Terentev et al. (2023)
	Optical Fluorescence Diagnostic	Firdous (2018)
	Remote Sensing Detection	Jing and Bai (2019)
	machine learning techniques	Azadbakht et al. (2019)
	Atomic Force Microscopy (AFM)	Firdous (2019)
	fluorescence sensors	Kuckenberg et al. (2009)
	RGB and thermal sensors	Zhou et al. (2015)
	UAV-based hyperspectral imaging	Zhang et al. (2019b)
	RGB and chlorophyll fluorescence	Bürling et al. (2011)
Stem rust	Drone Hyperspectral Imaging	Abdulridha et al. (2023)
	UAV and very high-resolution satellite imagery	Blasch et al. (2023)
Powdery Mildew	High-throughput imaging	Hinterberger et al. (2023)
	RGB imaging	Feng et al. (2016)
	“Macrobot”: An Automated Segmentation-Based System for Powdery Mildew Disease	Lück et al. (2020); Hinterberger et al. (2022)

As an example, one of the very useful phenotyping methods in wheat foliar disease is “Macrobot” (Lück et al., 2020). It was reported to be very helpful in phenotyping very-early stage of resistance of wheat powdery mildew after 24 and 72 hours of infection. The disease severity data generated from Macrobat was utilized to run GWAS which identified 51 significant markers associated with the resistance to powdery mildew (Hinterberger et al., 2022). Evaluating such a very-early stage of resistance is very helpful in mining genes controlling wheat powdery mildew in wheat. Therefore, HTP methods are precise and effective tools that can be used in genetic association analyses to accurately identify genomic regions associated with disease resistance in wheat, leading to the rapid genetic improvement of disease resistance. More efforts need to be made to integrate HTP methods in GWAS.

## 3 Genetics of foliar disease resistance in wheat

It has been widely known that the resistance of wheat yellow rust (WYR), wheat leaf rust (WLR), wheat stem rust (WSR), and wheat powdery mildew (WPM) follows two different genetic systems, seedling plant resistance (SPR, also known as all-stage resistance ASR) and adult plant resistance (APR). One main difference between these two types of wheat resistance is that ASR is a race-specific resistance that follows gene-by-gene theory (often called vertical resistance) and is expressed in early stage of wheat life cycle. While APR is often race none-specific resistance, therefore it is known as a durable resistance (also called horizontal resistance) that could not be easily broken by the changing in the pathogen race/s. However, APR, by definition, is not expressed in the early stage of wheat life cycle, leaving wheat seedlings in the danger of severe damage to early death in case of heavy infection. Therefore, the way to obtain highly ASR wheat genotypes is to pyramid many SPR/ASR and APR genes in the same genotype. This pyramiding was a goal of wheat breeders in the last decades. However, due to the continuous changes in the pathogen races, new races appear and break the resistance of known genes. Hence, identifying the new genes controlling the resistance of these new races is urgently needed. To date, a total of 84 WYR genes (Li et al., 2020a), more than 100 WLR genes (Kumar et al., 2022), over 66 WSR genes (Mago et al., 2022), and 68 WPM genes (McIntosh et al., 2020) were identified. Most of these identified resistance genes are race-specific and SPR.

Due to climate changes that leads to increasingly warmer weather, another important type of resistance should be considered, namely temperature sensitivity. Some of these known resistance genes were identified as temperature sensitive which means that these genes need specific range of temperature to work effectively. Furthermore, temperature sensitive resistance genes could be classified into genes work better in lower temperature such as *Sr15*, *Sr10*, Sr6 (effective when temperatures are less than 20°C), and genes work better in higher temperature (20-24°C) such as *Sr21*, *Sr23*, *Lr13*, *Yr18*, *Yr36*, *Yr52*, *Yr59*, *Yr62*, *Yr78*, *Lr67*/*Yr46*/*Sr55*, and *Yr79* [33,34]. For those resistance genes are more effective at higher temperatures, this type of resistance is known as high-temperature resistance (HTR). HTR was identified in adult growth stage, hence also known as high-temperature adult-plant (HTAP) resistance (Qayoum and Line, 1985). However, a novel type of resistance that was identified in the resistance of WYR disease is high-temperature all-stage resistance (HTAS) that was also found to be durable and race none-specific. Therefore, HTAS is also known as high-temperature seedling-plant resistance (HTSP) (Wang et al., 1995; Hu et al., 2023). Understanding the genetics of foliar disease resistance in wheat and detecting the required temperature for each gene will be more valuable for wheat breeders to obtain highly resistant wheat genotypes with the challenges of climate change.

### 3.1 Marker-assisted selection for foliar disease resistance

Marker-assisted selection (MAS) is still an important and essential method for genetically improving target traits in wheat. MAS identifies genomic regions associated with target traits in elite breeding lines, and is used in gene/trait pyramiding for quantitative traits. These genomic regions should be genetically validated before using them in MAS. Genetic validation of DNA markers tests whether the same QTL or gene is likely to be significantly associated with the target trait when the same population is grown in different years or/and other locations, as well as in other genetic backgrounds. A QTL that is effective in different environments and genetic backgrounds is more robust and reliable in breeding (Sallam et al., 2023).

Considerable research efforts have been performed to design specific DNA markers for specific genes for foliar disease. Most of the DNA markers were simple sequence repeats (SSR). The new advances in DNA technology provide Kompetitiv allele-specific PCR (KASP) markers which target a specific SNP that is associated with a specific gene. This SNP genotyping method has a lot of advantages over other DNA markers including high specificity and accuracy. Moreover, genotyping with KASP markers takes less time and effort, and is safer compared with handling other DNA markers. Therefore, using KASP markers for genotyping to target the resistant genotypes to foliar diseases in wheat will be very useful in MAS. Some of KASP markers that have been designed for specific rust resistance genes were reported previously in Bhavani et al., 2021 (Bhavani et al., 2021).

**Figure 1:**
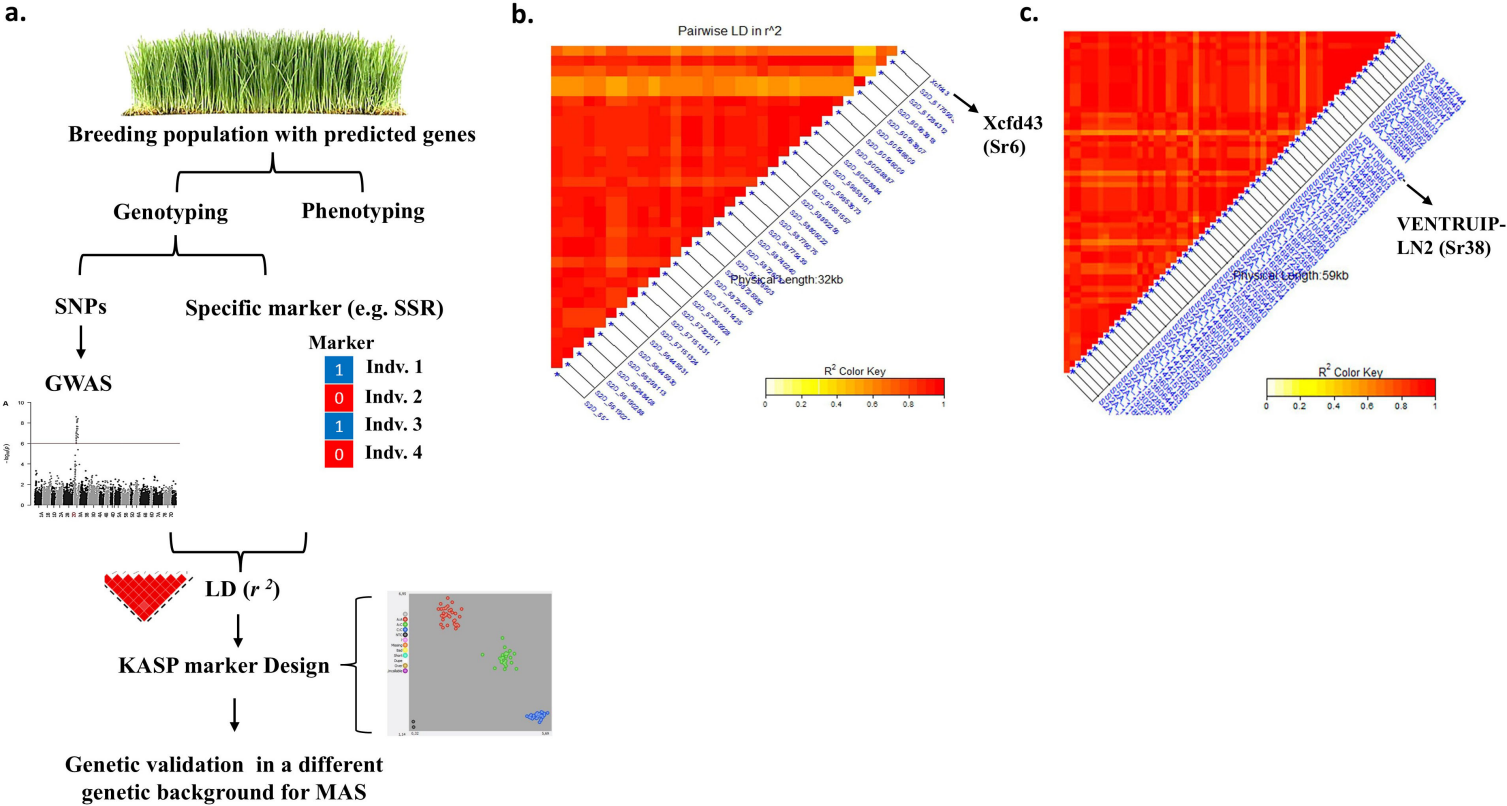
Graphical presentation showing **(A)** the usefulness of testing the LD between GWAS results and specific DNA markers for diease resistance to design KASP markers, **(B)** LD between Xcfd43 (Sr6) and SNP markers lcoated on 2D chromosome (Mourad et al 2018b), and **(C)** LD between VENTRUIPLN (Sr38) and SNP markers located on 2A chromosome (Eltaher et al., 2021).

Although KASP markers provide precise genotyping compared to other markers, the number of KASP compared to other markers is very low. Therefore, more research efforts are needed to detect SNPs for each resistance gene and then design the appropriate KASP marker for the target SNPs. Genome-wide association studies can cover this gap. Linkage disequilibrium (*r^2^*) can be performed between the high number of significant SNPs detected by GWAS and the alleles for specific markers (SSR, DArT, etc.) associated with the gene of interest. In this regard, Mourad et al. (2018b) reported 32 important candidate SNPs for the *Sr6* stem rust resistance gene by calculating LD between significant SNPs, detected by GWAS, located on the 2D chromosome and *Xcfd43* (specific marker for *Sr6* gene) marker allele (**Figure 1**). They performed GWAS on a set of 270 winter wheat genotypes and they verified the high LD between these SNPs and the *Xcfd43* marker allele in an additional 60 wheat genotypes belonging to different genetic backgrounds. The R^2^ of the 32 SNPs ranged from 10.99 to 17.68% which indicated a major genomic region. Therefore, designing a KASP marker for any of these SNPs that was in a completely significant LD with the *Xcfd453* marker allele could be used for investigating the presence of the *Sr6* gene in the target population. Using the same approach, Eltaher et al., 2021 (Eltaher et al., 2021) tested the LD between all significant SNPs (65 markers) located on chromosome 3A and VENTRIUP-LN2 (specific marker for *Sr38* gene) marker alleles in 184 winter wheat genotypes. The authors found a highly significant LD between 59 SNPs and the VENTRIUP-LN2 allele (**Figure 1**). The R^2^ of these 59 SNPs ranged from 13.68 to 25.68%, indicating a major genomic region associated with stem rust resistance. Two important factors documenting the robustness of this association were (1) *Xcfd453* (*Sr6*) and VENTRIUP-LN2 (*Sr38*) alleles were highly significant associated with stem rust resistance in the two populations with a *p*-value of <0.0001 (Mourad et al., 2018b) and 2.45 × 10^-33^ (Eltaher et al., 2021), respectively, and (2) both studies predicted the presence of these two genes in their breeding material. In this research using plant material that is expected to carry a few specific genes will be most useful and easier to identify candidate SNPs for the target genes. The complexities of pyramided genes and epistasis make identifying markers for specific genes in these lines more difficult. Once the association between the SNPs, detected by GWAS, and the specific marker allele for the target genes is confirmed, a KASP marker can be designed and validated in a different genetic background before using it in the MAS.

### 3.2 Recent genome-wide association studies on foliar disease resistance

With the recent advances in genome sequencing methods, GWAS became a powerful tool in detecting the genomic regions controlling different targeted traits. It has been used widely due to its simplicity, dense maker genome coverage, and power in detecting new genomic regions associated with the targeted trait. GWAS was reported as an effective tool in detecting new genomic regions that have not been detected using QTL method using biparental populations (Alqudah et al., 2020). A large number of GWAS studies have been done to understand the genetic control of foliar disease resistance in wheat. However, due to the continuous changes in the virulence ability of the pathogen, some of the detected genomic regions lost their effectiveness. Therefore, in this review, we focused on the GWAS studies that have been done in the last five years (from 2019 to the mid of 2023) to summarize genomic regions that are more likely still effective against the most recent pathogen races.

**Figure 2:**
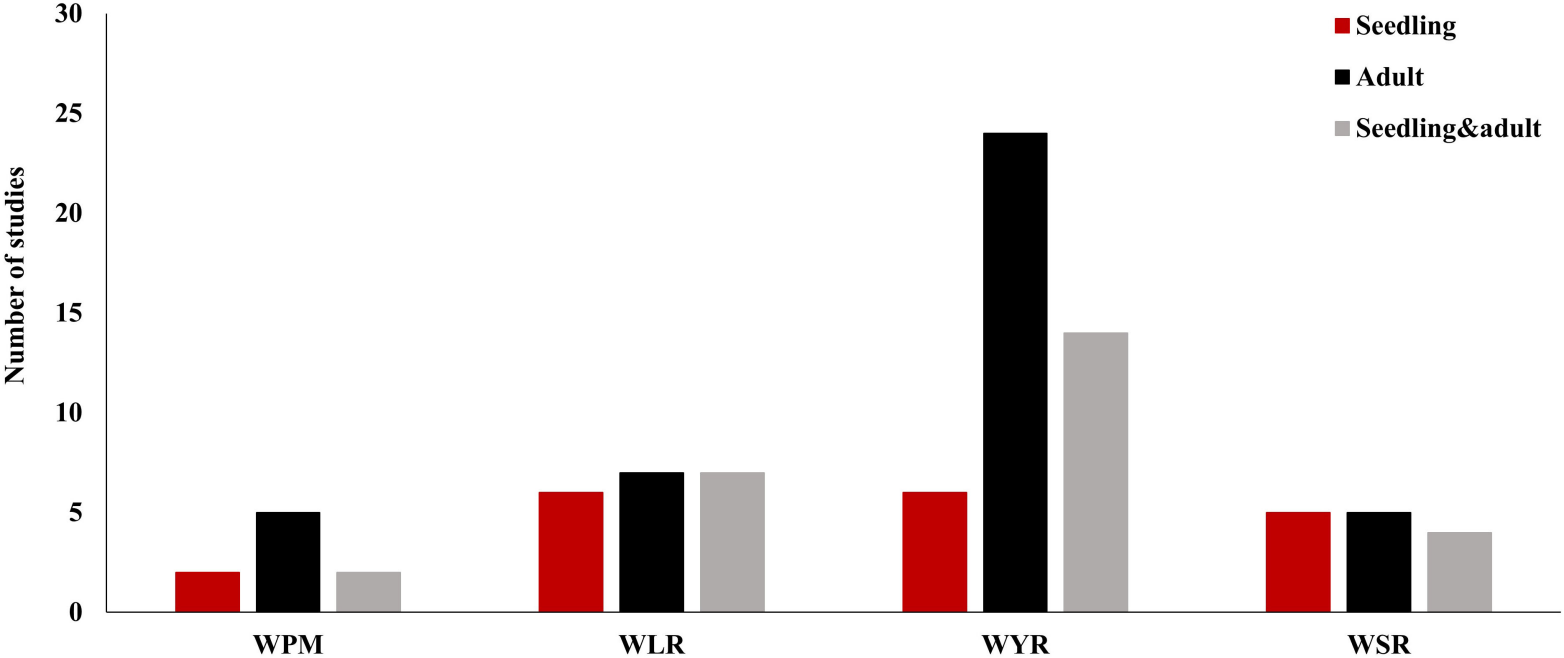
Number of GWAS studies carried out for wheat powdery mildew (WPM), wheat leaf rust (WLR), wheat yellow rust (WYR), and wheat stem rust (WSR) in different growth stages of wheat life cycles.

The highest number of GWAS studies (44) were done to understand the genetic control of WYR (Ledesma-Ramírez et al., 2019; Liu et al., 2019, 2020b; Long et al., 2019; Nyine et al., 2019; Yao et al., 2019, 2020, 2021; Ye et al., 2019; Cheng et al., 2019, 2020; Genievskaya et al., 2020; Jia et al., 2020; Juliana et al., 2020; Kumar et al., 2020; Li et al., 2020b; Miedaner et al., 2020a; Mu et al., 2020; Pradhan et al., 2020; Wu et al., 2020; Yang et al., 2020, 2023; Beukert et al., 2020; Abou-Zeid and Mourad, 2021; Alemui et al., 2021; Aoun et al., 2021; Morales et al., 2021; Tehseen et al., 2021; Tomar et al., 2021; Wang et al., 2021b; Ward et al., 2021; Zhang et al., 2021; Brandt et al., 2021; Franco et al., 2022; Iqbal et al., 2022; Jambuthenne et al., 2022; Mahmood et al., 2022; Saleem et al., 2022b; Baranwal et al., 2022; Shahinnia et al., 2022, 2023; El Messoadi et al., 2022; Klymiuk et al., 2023; Esmail et al., 2023b) (**Figure 2** and **Supplementary Table S1**). This level of research was expected due to the devastating effect of this disease on wheat production around the world. Out of these studies, 24 focused on the adult plant growth stage while only six studies investigated the seedling growth stage resistance. Fourteen studies focused on the resistance at both growth stages (seedling and adult). Therefore, we can conclude that WYR got most of wheat breeders’ attention in the last five years. However, more attention should be given to understand the seedling resistance to WYR. Both WLR and WSR had a similar the number of GWAS studies was almost the same for both diseases, 20 and 14, respectively (Mourad et al., 2019, 2022; Sapkota et al., 2019; Beukert et al., 2020; Leonova et al., 2020b, 2020a; Liu et al., 2020a; Megerssa et al., 2020, 2021; Miedaner et al., 2020a; Fatima et al., 2020; Genievskaya et al., 2020, 2022; Kumar et al., 2020; Saremirad et al., 2021; Zhang et al., 2021; Eltaher et al., 2021; Baranwal et al., 2022; Negash et al., 2022; Saleem et al., 2022a; Shewabez et al., 2022; Tyagi et al., 2022; Vikas et al., 2022; Zatybekov et al., 2022; Iqbal et al., 2022; Lhamo et al., 2023; Delfan et al., 2023; Talebi et al., 2023; Klymiuk et al., 2023) (**Figure 2** and **Supplementary Tables S2**, **S3**). In these studies, wheat breeders paid the same attention to both seedling and adult plant resistance for these diseases. The lowest number of GWAS studies for foliar wheat diseases (nine studies) was drawn to WPM (Kang et al., 2019; Mohler and Stadlmeier, 2019; Miedaner et al., 2020b; Simeone et al., 2020; Alemu et al., 2021; Du et al., 2021; Hinterberger et al., 2022; Jin et al., 2022; Mourad et al., 2023) (**Figure 2** and **Supplementary Table S4**). WPM has been reported as one of the major devasting foliar diseases in wheat and has caused up to 62% yield losses (Costamilan, 2005; Maxwell et al., 2009; Huang et al., 2013). Some recent studies identified novel genomic regions controlling the resistance of this disease (Mourad et al., 2023). Therefore, more GWAS research studies are needed to unravel the genetic control of this serious disease. Interestingly, the majority of GWAS studies conducted on each foliar disease were done at only one growth stage (seedling or adult). Combining both growth stages in GWAS studies will be more helpful in understanding the genetic control of resistance at both growth stages. Hence enable wheat breeders to gather all these genomic regions in few genotypes and produce a high level of resistance in the same genotype across all growth stages. To achieve this goal, more concern should be given to seedling growth stage resistance especially for WYR and WPM.

**Figure 3:**
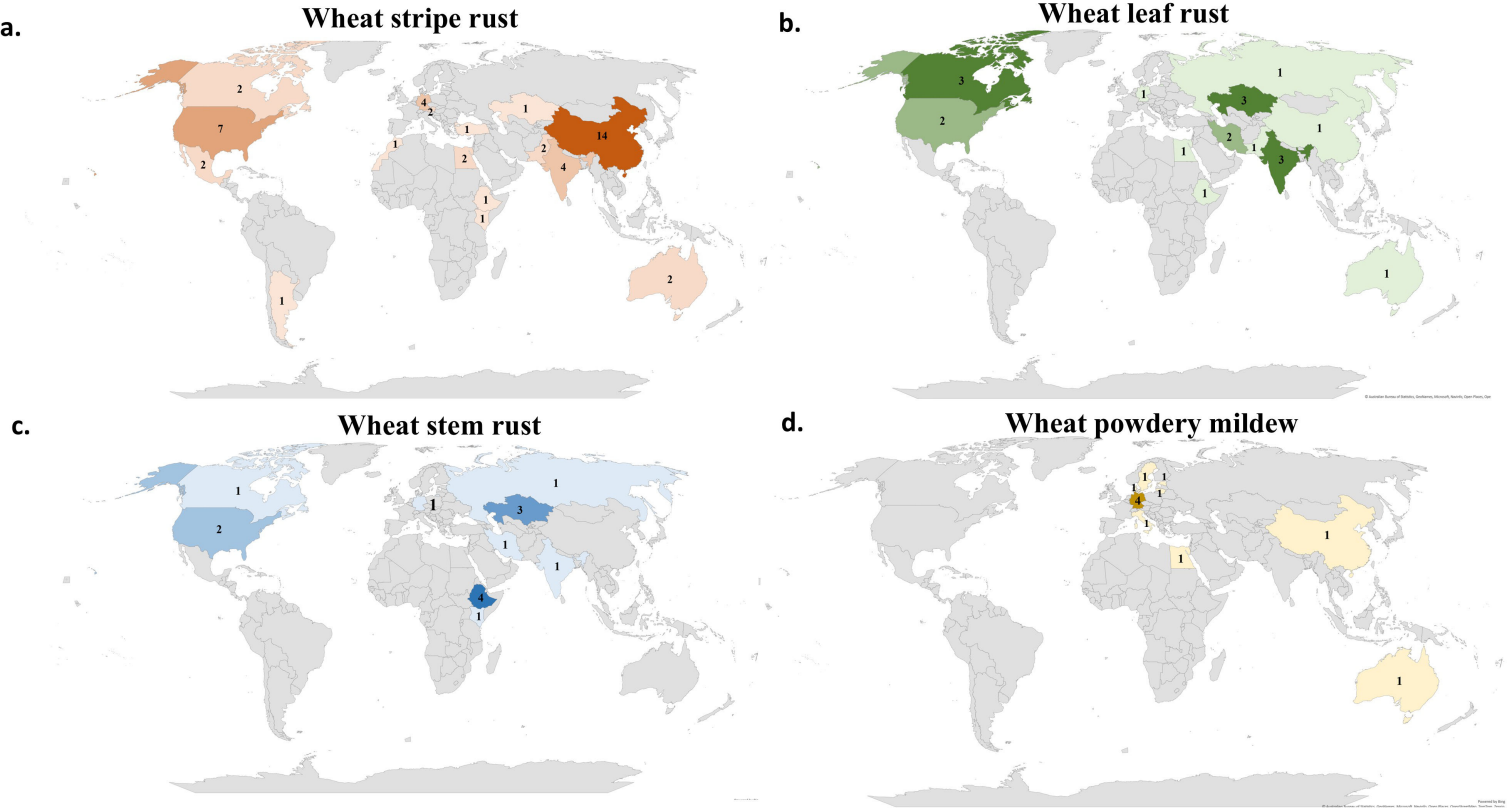
Number of GWAS studies carried out for; **(A)** wheat yellow rust (WYR), **(B)** wheat leaf rust (WLR), **(C)** wheat stem rust (WSR) and **(D)** wheat powdery mildew (WPM) in different countries of the world.

The four studied foliar diseases affect wheat planting areas around the world. In each region of the world there are different races from the pathogen. Therefore, it is very important to know the geographical regions and pathogen complexes where the identified genomic region/s was/were detected. In the last five years, the majority of WYR studies were done in China (14 studies) followed by the U.S.A (seven studies) (**Figure 3A**). Interestingly, Europe and Africa had very few numbers of GWAS studies for WYR with a total number of seven and five studies, respectively. South America has only one study that was carried out in Argentina. Despite the large area of Canada that guarantee the presence of many *Pst* races, only two GWAS studies have been done. Many countries did not conduct any GWAS studies in the last five years for such a serious disease including some developed countries such as U.K., Finland, and Denmark. Therefore, we can conclude that, despite the high number of GWAS studies carried out on WYR, more efforts should be made to identify the most important genomic regions controlling the resistance. The low number of GWAS studies in some regions keeps the genetic control against some *Pst* races unknown, hence hinders the genetic improvement of WYR resistance in growing wheat cultivars. Unlike WYR, WLR and WSR had a lower number of GWAS studies that were distributed among the three old continents. Asia had the highest number of GWAS studies for both WLR and WSR with a number of nine and six studies, respectively (**Figures 3B, C**). Noth America and Africa occupied the second place for the GWAS studies on WLR and WSR respectively with five studies for each disease. Surprisingly, Europe has only one GWAS study for WLR and WSR. The lowest number of WLR studies were found in Africa as one study was carried out in Egypt (**Figure 3B**). As it was mentioned previously, very few efforts have been made to detect the genetic control of WPM resistance using GWAS. Most of these efforts were done in Europe as six studies were done among different European countries. Only one GWAS study was done in Australia, China, and Egypt, respectively (**Figure 3D**). Interestingly, no WPM GWAS study was done in North or South America.

In summary, the recent GWAS efforts should be dramatically increased to obtain effective broad-spectrum resistance. A large number of wheat genotypes should be evaluated for their resistance in different growth stages as well as in different parts of the world. This will enable the detection of important genomic regions controlling the resistance against different races of fungal pathogens.

## 4 Potential genetic markers associated with broad-spectrum resistance for each foliar disease

Due to the low number of GWAS studies that have been done in the last five years, few markers were found to be common among different regions. This confirms the need of increasing GWAS studies to enhance wheat breeding programs by identifying the stable genomic regions controlling the resistance to different races of the disease pathogen.

**Figure 4:**
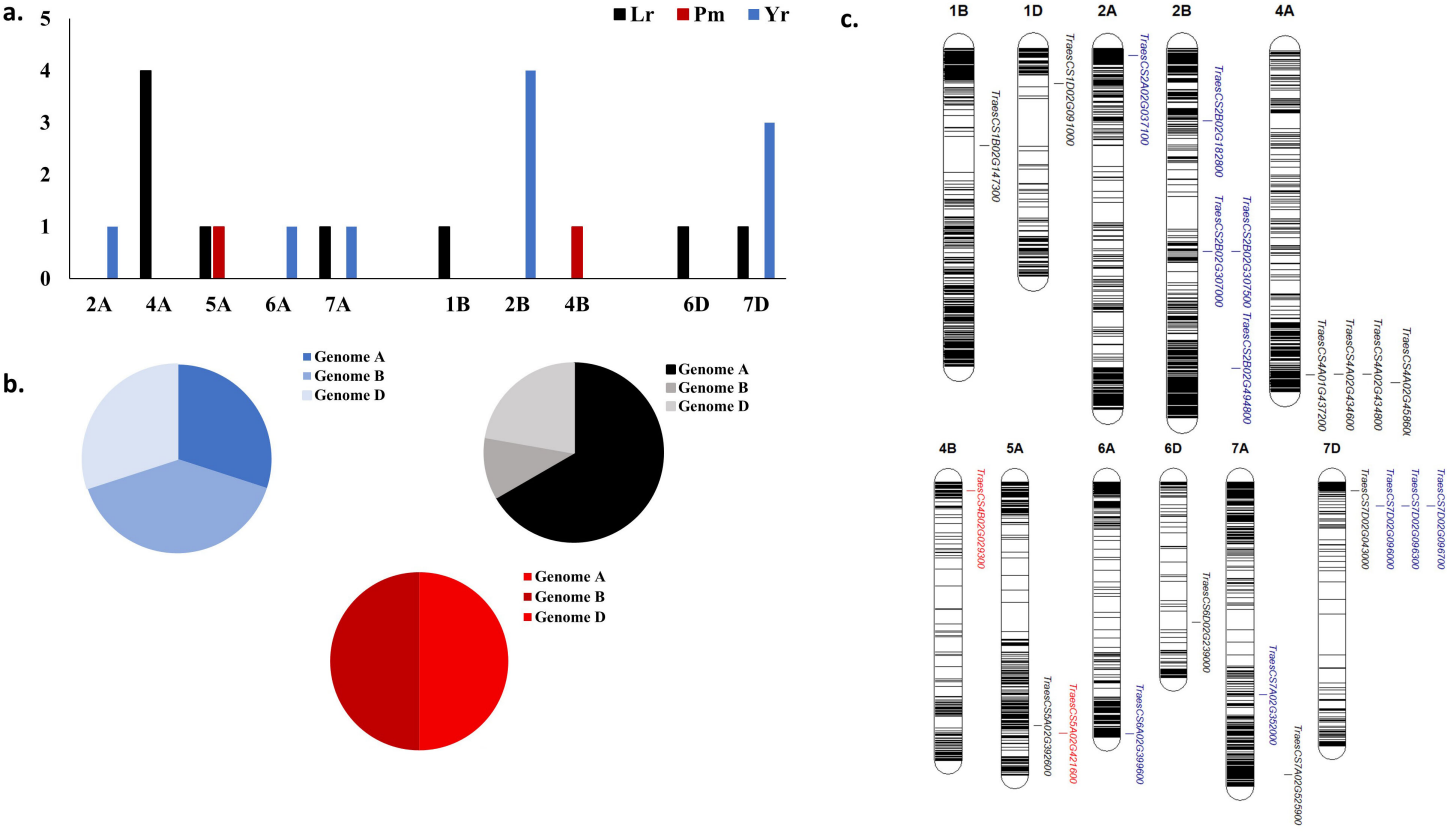
Distribution of common gene models associated with each studied foliar disease among different regions around the world, **(A)** number of markers associated with each disease in each chromosome, **(B)** distribution of significant markers associated with each disease in each wheat genome, and **(C)** chromosomal position of gene models harboring these common significant markers.

**Figure 5:**
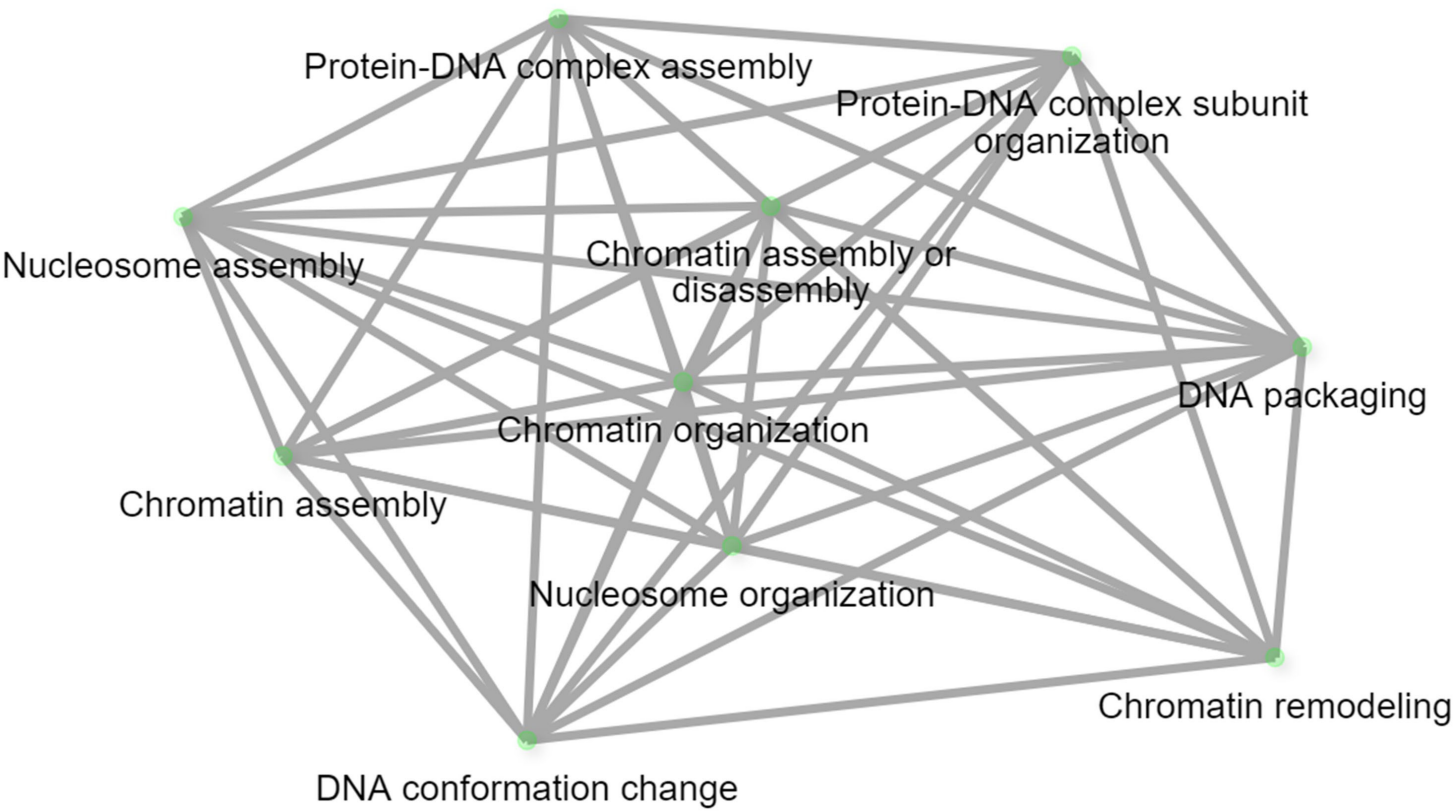
WYR biological process network.

The results of the 44 GWAS studies reported 9,346 markers associated with the resistance against different *Pst* races around 16 different countries (**Supplementary Table S1**). Out of the 9,346 markers, only 14 were found to be commonly associated with WYR resistance in at least two different countries (**Table 2**). These markers were found to be distributed among seven chromosomes and located within ten different gene models. The ten gene models harboring these significant markers were distributed among five different chromosomes with a total of four genes on chromosome 2B, three genes on 7D and one gene on each of 2A, 6A, and 7A chromosomes (**Figure 4A**). Genome B carried the highest percentage of broad-spectrum gene models with a percentage of 40% followed by genome A and B (30%) (**Figure 4B**). Notably, the three gene models on chromosome 7D were located near to each other on the short arm of the chromosome indicating the presence of an important genomic region associated with the resistance under German and Canadian conditions (**Figure 4C**) (Iqbal et al., 2022; Shahinnia et al., 2023). However, the four gene models on chromosome 2B were distributed across the chromosome and associated with the resistance against *Pst* races in Germany, Austria, Egypt, and Canada. Therefore, chromosome 2B seems to contain many different genomic regions that contribute to WYR broad-spectrum resistance against Warrior (-) FS 53/20, 78E159, 174E191 and 246E175 *Pst* races under Germany, Austria, Egypt, and Canada environmental conditions (Beukert et al., 2020, 2021; Iqbal et al., 2022; Shahinnia et al., 2022, 2023; Esmail et al., 2023b). To provide more understanding of the role of these ten gene models in providing broad-spectrum resistance against different *Pst* races, the functional annotation of these genes was detected based on IWGSC and presented in **Table 2**. Two genes, *TraesCS7D02G096000* and *TraesCS2B02G182800*, were found to control NLR-disease resistance protein and RPM1. These proteins were reported to control WYR seedling resistance under high temperature conditions (Wang et al., 2020). Based on the recent reviewed studies, RPM1 was significantly associated with WYR resistance not only at seedling growth stage but also at seedling and adult growth stages (Iqbal et al., 2022; Shahinnia et al., 2022, 2023). The remaining gene models were found to control the production of other proteins and enzymes. However, we looked for the gene enrichment of the identified gene models using ShinyGo 0.77 database (Ge et al., 2022) (1% FDR). All the identified gene models were found to work together in one network and control different biological process pathways that complementary control disease resistance in wheat such as chromatin organization and regulation of DNA expression (**Figure 5**). Chromatin organization and DNA expression regulation were reported previously to be included in defense regulation components involved in wheat resistance against stripe rust (Andersen et al., 2020). Therefore, more concern should be given to better understanding about the identified ten gene models and their role in broad-spectrum resistance against WYR.

**Table 2:** List of significant markers associated with wheat yellow rust (WYR) resistance among different regions, their chromosomal position (bp), year of evaluation, *Puccinia striiformis* f.sp*. tritici (Pst)* races that they resist, and gene models they are located within.

Marker	Chr	Position (bp)	Evaluation year	Wheat type	Growth stage	Races	Regions	Gene models	Citation	Functional annotation*
BobWhite_c40479_283	7D	58258742	2018	Spring,winter	Seedling, adult	*Warrior (-)* G 23/19	Germany, Canada	NA	Iqbal et al. (2022); Shahinnia et al. (2023)	–
BS00093016_51	7A	515199467	2020-2022	Spring,winter	Seedling, adult	78E159, 174E191, 246E175	Egypt, Germany Austria	*TraesCS7A02G352000*	Shahinnia et al. (2022); Esmail et al. (2023b)	Peroxidase
CAP12_c259_307	2A	15875830	2016-2017 2020-2021	Spring,winter	Adult	NA	Germany, Morocco	*TraesCS2A02G037100*	Beukert et al. (2020); El Messoadi et al. (2022)	serine-type endopeptidase
D_GDS7LZN02FSYZC_227	7D	58491701	2018	Winter,spring	Seedling, adult	*Warrior (-)* G 23/19	Germany, Canada	TraesCS7D02G096000*	Iqbal et al. (2022); Shahinnia et al. (2023)	Disease resistance protein RPM1
IAAV1743	2B	439225308	2016-2017 2020-2021	Winter	Seedling, adult	*Warrior (-) FS 53/20*	Germany, Austria	TraesCS2B02G307000*	Beukert et al. (2020); Shahinnia et al. (2022, 2023)	Protein phosphatase 2C containing protein
IAAV6424	2B	691780764	2016/2022	Spring	Seedling, adult	78E159, 174E191, 246E175	Canada, Egypt	*TraesCS2B02G494800*	Iqbal et al. (2022); Esmail et al. (2023b)	Leguminosin group485 secreted peptide
IWA1787	1D	8.6 (Mb)	2016/2019	Winter	Adult	CYR32, CYR37	China, USA	NA	Jia et al. (2020); Mu et al. (2020)	–
Kukri_c32845_116	7D	58504378	2018	Winter,spring	Seedling, adult	*Warrior (-)* G 23/19	Germany, Canada	TraesCS7D02G096300*	Iqbal et al. (2022); Shahinnia et al. (2023)	Nucleosome assembly protein
Kukri_c92151_216	7D	58485512	2018	Winter,spring	Seedling, adult	*Warrior (-)* G 23/19	Germany, Canada	NA	Iqbal et al. (2022); Shahinnia et al. (2023)	–
Ra_c6266_136	2B	440214889	2016-2017 2020-2021	Winter	Seedling, adult	*Warrior (-) FS 53/20*	Germany, Austria	TraesCS2B02G307500*	Beukert et al. (2020); Shahinnia et al. (2022, 2023)	ATP-dependent Clp protease
RAC875_c1226_652	2B	157693607	2016-2017 2020-2021	Winter	Adult	NA	Germany, Austria	TraesCS2B02G182800*	Beukert et al. (2020); Shahinnia et al. (2022)	NBS-LRR disease resistance protein
TA002254-0660	7B*	7BL_4601295*	2016-2017	Spring,winter	Adult	NA	Canada, Germany	NA	Beukert et al. (2020); Iqbal et al. (2022)	–
TA002473-0717	7D	58644217	2018	Winter,spring	Seedling, adult	*Warrior (-)* G 23/19	Germany, Canada	TraesCS7D02G096700*	Iqbal et al. (2022); Shahinnia et al. (2023)	Cysteine synthase
Tdurum_contig29607_413	6A	609380034	2016-2017 2020-2021	Winter	Adult	NA	Germany, Austria	TraesCS6A02G399600*	Beukert et al. (2020); Shahinnia et al. (2022)	RING/U-box superfamily protein

*Refers to chromosomal position and gene models obtained from *Ensembleplants* database by the authors and don’t published in the reference article.

Based on the 20 GWAS studies on WLR resistance in 12 different countries, a total of 2,586 markers were identified to be significantly associated with WLR resistance (**Supplementary Table S2**). Out of these markers, 11 were identified to be significantly associated with WLR resistance in at least two different countries (**Table 3**). These markers were found to be located on seven different chromosomes and located within eleven different gene models (**Table 3** and **Figure 4A**). The highest number of gene models harboring significant markers were located on chromosome 4A with four genes. The remaining genes were distributed among 5A, 7A, 1B, 6D, and 7D chromosomes with one marker on each chromosome. The highest percentage of gene models were located in genome A (67%) followed by genome D (22%) then genome B (11%) (**Figure 4B**). The four gene models located on 4A chromosome were found to locate near to each other on the long arm of the chromosome, thus major genomic region controlling WLR under Chinese and German conditions is expected on this chromosome. Unfortunately, the *Pt* races were not mentioned in these studies (Beukert et al., 2020; Fatima et al., 2020; Zhang et al., 2021). Furthermore, *TraesCS7A02G525900* gene model was found to control the resistance of six different *Pt* races at seedling growth stage in Egypt and Ethiopia (Mourad et al., 2022; Lhamo et al., 2023). Based on the IWGSC, the functional annotation of this gene was found to control zinc finger protein that was reported to improve wheat tolerance and resistance to abiotic and biotic stresses (Han et al., 2021) (**Table 3**). The same function was found to be produced by *TraesCS4A02G434600* and *TraesCS4A02G434600* genes that controlling WLR adult plant resistance in China and Germany suggesting the potential importance of zinc finger protein in producing broad-spectrum resistance against different races of *Pt* pathogen. The existence of Zinc finger protein domains was reported in diseases resistance proteins of nine different crops including Arabidopsis, barley, flax, potato, rice, sunflower, tomato, tobacco, and wheat (Gupta et al., 2012). Unfortunately, no available information exists on the gene enrichment of the other ten identified gene models. However, based on the functional annotation of these gene models, all these genes contribute to fungal disease resistance in plants (Feuillet et al., 1997; Andersen et al., 2020; Sun et al., 2022; Laribi et al., 2023; Oladzad et al., 2023). Therefore, the identified gene models and their significant markers could be used in producing wheat genotypes that have a wide range of WLR broad-spectrum resistance as well as in MAS of this trait. The 14 GWAS studies identified 889 markers associated with WSR resistance in ten different countries. However, none of the identified markers were associated with WSR resistance in different countries. Therefore, no markers were identified to be associated with WSR broad-spectrum resistance.

**Table 3:** List of significant markers associated with wheat leaf rust (WLR) resistance among different regions, their chromosomal position (bp), year of evaluation, *Puccinia triticina (Pt)* races that they resist, and gene models they are located within.

Marker	Chr	Position (bp)	Evaluation year	Wheat type	Growth stage	Races	Regions	Gene models	Citation	Functional annotation*
BS00084703_51	4A	705508415	2015-2017	Winter	Adult	NA	China, Germany	TraesCS4A02G434800*	Zhang et al. (2021); Beukert et al. (2020)	Kinase-like protein
BS00091561_51	4A	705178373	2015-2017	Winter	Adult	NA	China, Germany	TraesCS4A02G434600*	Zhang et al. (2021); Beukert et al. (2020)	zinc finger B-box protein
D_GB5Y7FA02G6KBX_382	6D	339399173	2016-2018	Spring, winter	Seedling, adult	MBRJ/NA	Canada, Russia	*TraesCS6D02G239000*	Fatima et al. (2020); Leonova et al. (2020b)	tRNA-specific 2-thiouridylase MnmA 1
Excalibur_c13811_1086	4A	723810464	2016-2019	Spring, winter	Adult	NA	Canada, Germany	*TraesCS4A02G458600*	Fatima et al. (2020); Beukert et al. (2020)	RNA-binding protein
IAAV2452	1A^$^\1D*	77367932-77368066*	2016-2018	Spring	Seedling, adult	FGRQ/NA	China, Russia	*TraesCS1A02G109300*/TraesCS1D02G091000*	Zhang et al. (2021); Leonova et al. (2020b)	Pentatricopeptide repeat superfamily protein
IAAV6025	1B	211312383	NA	Spring, winter	Seedling	FGRQ, BDS, MBRJ	China, Canada	*TraesCS1B02G147300*	Zhang et al. (2021); Fatima et al. (2020)	Trichome birefringence-like protein
IACX8322	7D*	22223068-22223209*	2015-2017	Winter	Adult	NA	Germany, China	TraesCS7D02G043000*	Beukert et al. (2020); Zhang et al. (2021)	Superoxide dismutase
Kukri_c17055_189	5A	588742167	NA	Spring, winter	Seedling	TDBG2, FGRQ	Canada, China	*TraesCS5A02G392600*	Fatima et al. (2020); Zhang et al. (2021)	ABC transporter B family protein
Tdurum_contig93100_149	4A	705178373	2015-2017	Winter	Adult	NA	China, Germany	TraesCS4A02G434600*	Zhang et al. (2021); Beukert et al. (2020)	zinc finger B-box protein
wsnp_Ex_c4331_7808746	4A	707042951	2016-2017	Winter	Adult	NA	China, Germany	*TraesCS4A01G437200*	Zhang et al. (2021); Beukert et al. (2020)	Protein ENHANCED DISEASE RESISTANCE
wsnp_Ku_c5693_10079278	7A	708143182	2013-2014 /2020	Spring	Seedling	LGDTT, HTTDS, PBPPP, BBBQJ, CCMSS and MCDSS	Egypt, Ethiopian	*TraesCS7A02G525900*	Mourad et al. (2022); Lhamo et al. (2023)	Zinc finger CCCH domain protein

*Refers to chromosomal position and gene models obtained from *Ensembleplants* database by the authors and don’t published in the reference article.

^$^Different chromosomal positions mean that this is homologous gene.

The nine GWAS studies carried out on WPM resistance reported the presence of 733 significant markers associated with the resistance in nine different countries (**Supplementary Table S4**). Out of these significant markers, two markers were found to be significantly associated with the resistance in at least two different countries (**Table 4**). Both markers were associated with the resistance in spring and winter wheat at both seedling and adult growth stage. Marker Kukri_c6266_260 located within *TraesCS5A02G421600* gene model was associated with the resistance in Egypt, Lithuania and Estonia. It was functionally annotated to control the production of S-adenosyl-L-methionine-dependent methyltransferases superfamily protein, a protein that was reported to play an important role in methyl groups transference and disease development (Struck et al., 2012). Furthermore, marker Kukri_c52413_282 located with *TraesCS4B02G029300* gene model was associated with the resistance in Egypt and Sweden. It was found to be functionally annotated to control the production of NADH dehydrogenase (**Table 4**). The reduction in NADH dehydrogenase activity was reported to increase wheat plants resistance to fungal diseases and insect infestation (Bai et al., 2022). The identified two gene models seem to play an important role in providing broad-spectrum resistance against different races from *Bgt* in Egypt and Europe. However, more studies should be done to identify the genetic control of WPM around the world.

**Table 4:** List of significant markers associated with wheat powdery mildew (WPM) resistance among different regions, their chromosomal position (bp), year of evaluation, *Blumeria graminis* f. sp. *tritici* (*Bgt*) races that they resist, and gene models they are located within.

Marker	Chr	Position (bp)	Evaluation year	Wheat type	Growth stage	Races	Regions	Gene models	Citation	Functional annotation
Kukri_c6266_260	5A	607682915	2019-2021	Spring, winter	Seedling, adult	NA	Egypt, Lithuania, Estonia	*TraesCS5A02G421600*	Mourad et al. (2023); Alemu et al. (2021)	S-adenosyl-L-methionine-dependent methyltransferases superfamily protein
Kukri_c52413_282	4B	21553917	2019/2021	Spring, winter	Seedling, adult	NA	Egypt, Sweden	*TraesCS4B02G029300*	Mourad et al. (2023); Alemu et al. (2021)	NADH dehydrogenase

The previously discussed studies provided important information on the genetic control of broad-spectrum resistance of four important foliar diseases affecting wheat planting areas around the world. The identified common SNP markers could be used in MAS for the resistance. Based on the provided information, the genetic role of the identified gene models was roughly understood. However, more studies should be done to provide more understanding of the genetic role of broad-spectrum resistance. Hence, producing wheat genotypes that are highly resistance to each disease.

## 5 Potential genetic markers associated with different foliar diseases

**Figure 6:**
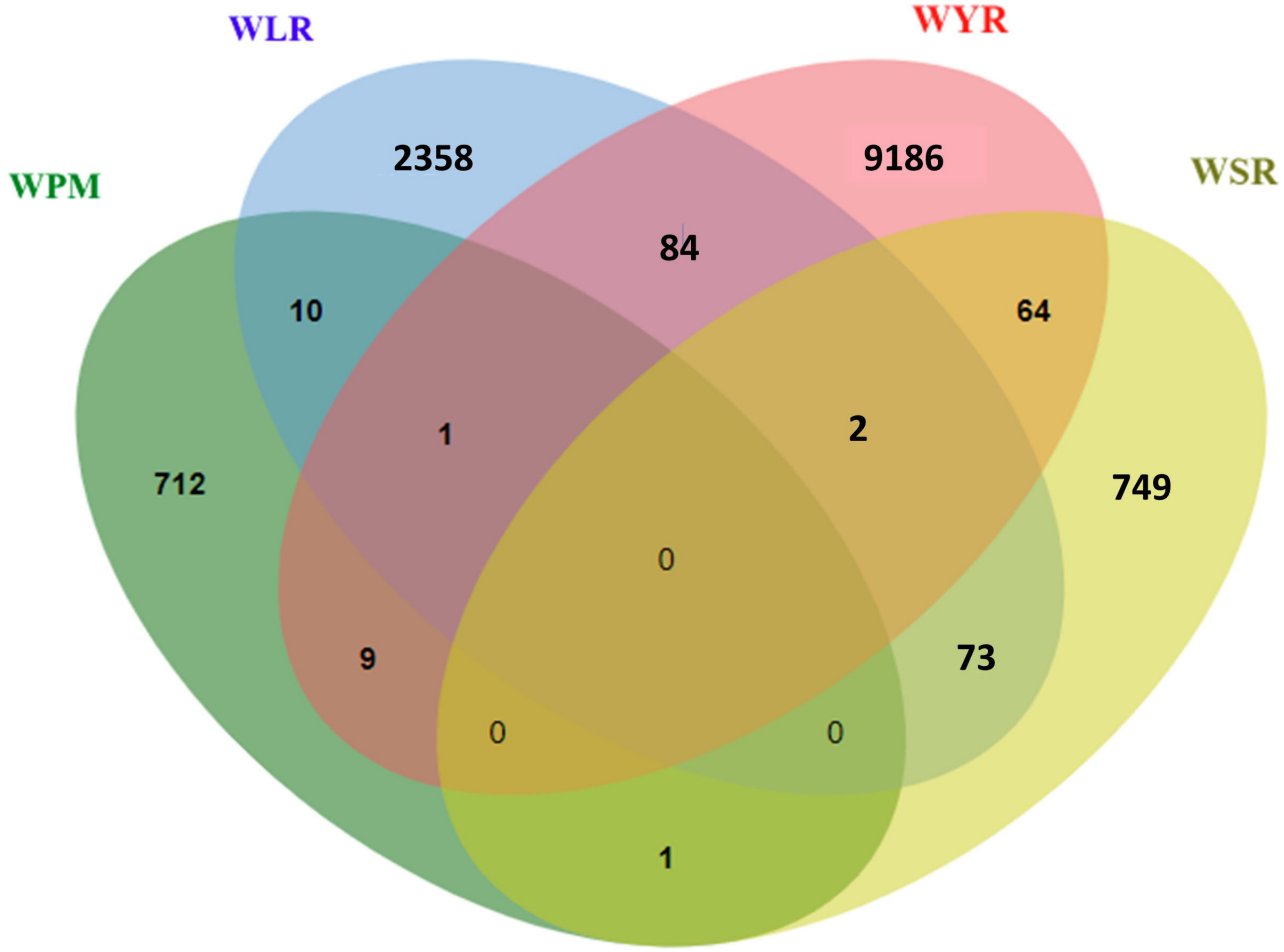
Number of significant markers associated with the resistance of the four studied foliar diseases from 2019 to 2023.

The four diseases studied in the current review are common in their infection nature as they infect the foliar parts of wheat plants. However, the wheat genome is a highly complex one. Therefore, breeding for disease resistance would be more effective if a common mechanism is found to control the resistance of the four foliar diseases. This common resistance will help the wheat breeder and simplify his job in producing genotypes with high levels of resistance against several foliar diseases. Therefore, we looked for common markers associated with the resistance of at least two diseases. Out of the identified markers significantly associated with each disease, one marker was common between WPM, WYR, and WLR, one marker between WPM and WYR, and ten markers between WPM and WLR, respectively (**Figure 6**). Furthermore, two markers were commonly associated with the resistance of the three rust diseases, 84 markers between WLR and WYR, 64 between WYR and WSR, and 73 between WLR and WSR, respectively.

**Figure 7:**
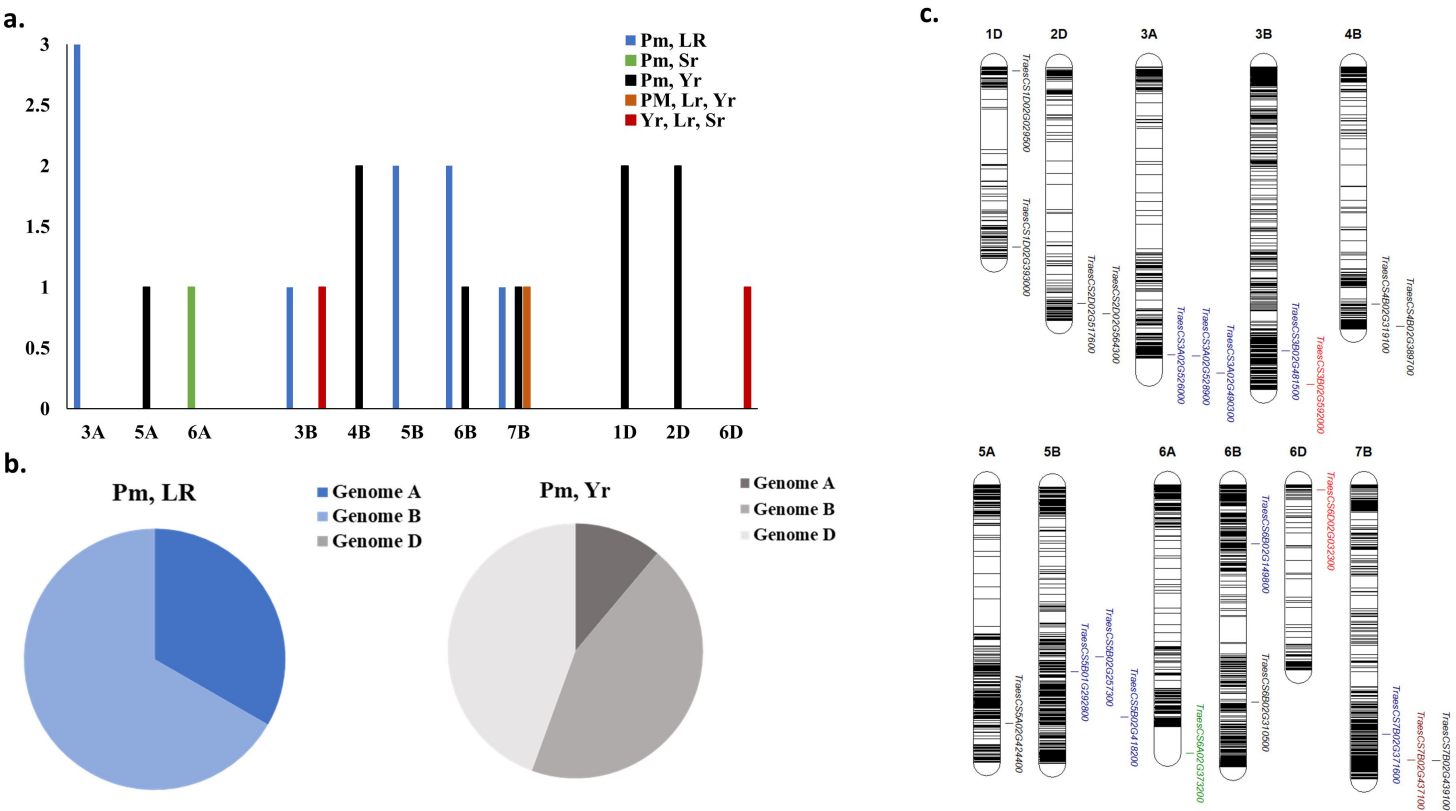
Distribution of common gene models associated with more than one foliar disease; **(A)** number of gene models in each chromosome, **(B)** distribution of these gene models each wheat genome, and **(C)** chromosomal position of gene models.

Common markers associated with WPM and one of the rust diseases were distributed among ten different chromosomes with a number of three markers on chromosome 3A, 6B, and 7B, two markers on each of 4B, 5B, 2D and 2D chromosomes, and one marker on each of 5A, 6A, and 3B (**Figures 7A, B**). However, the two markers associated with the three rust diseases were located on 3B and 5D with one marker on each chromosome.

Marker that was commonly associated with WPM, WLR, and WYR was located on chromosome 7B and within *TraesCS7B02G437100* gene model. It was associated with WPM adult plant resistance in Estonia and seedling resistance of both WLR and WYR in Canada and Germany, respectively (**Table 5**). The phenotypic variation explained by this marker (R^2^) was lower than 10% for all the three diseases indicating the minor effect of this marker on the resistance of these diseases (**Supplementary Tables S1–S3**, **S9**). The functional annotation of this gene was controlling Transcriptional corepressor SEUSS production, a transcription repressor that induced in response to auxin signaling and regulates developments traits (Sari et al., 2019). The same function was found to be over expressed under fusarium head blight (FHB, *Fusarium graminearum*) infection in wheat. Therefore, this gene and its associated marker could be used as MAS for the resistance of several wheat diseases in wheat.

**Table 5:** List of significant markers associated with wheat powdery mildew (WPM) resistance and at least one rust disease, their chromosomal position (bp), year of evaluation, region of evaluation, races that they resist, and gene models they are located within.

Marker	Chr	Position (bp)	Gene models	Foliar disease	Evaluation year	Wheat type	Growth stage	Races	Regions	Citation	Functional annotation
Tdurum_contig67161_99	7B	703310984	TraesCS7B02G437100*	WPM	2019	Winter	adult	NA	Estonia	Alemu et al. (2021)	Transcriptional corepressor SEUSS
				WLR	NA	Winter & spring	Seedling	TJBJ	Canada	Fatima et al. (2020)	
				WYR	NA	winter	Seedling	NA	Germany	Shahinnia et al. (2023)	
IWB74	2D	741856706-741856806	*TraesCS2D02G517600*	WPM	2017	NA	Adult	S509 and T509	Australia	Kang et al. (2019)	Ubiquitin carboxyl-terminal hydrolase
				WYR	2015	NA	Adult	CYR32, CYR33, CYR34	China	Yang et al. (2020)	
IWB10765	5A	609870594-609870494	*TraesCS5A02G424400*	WPM	2018	NA	Adult	S509 and T509	Australia	Kang et al. (2019)	Peroxisomal (S)-2-hydroxy-acid oxidase
				WYR	2019	NA	Adult	145 E16A + Yr17 + Yr27, 150 E16A + 134 E16A + YrJ + YrT + and 239 E237A-Yr17 + Yr33 +)	Australia	Baranwal et al. (2022)	
RAC875_c1357_860	4B	609498171	TraesCS4B02G319100*	WPM	2019	Winter	Adult	NA	Denmark	Alemu et al. (2021)	Chaperone protein dnaJ
				WYR	2020	Winter	Adult	NA	Germany	Shahinnia et al. (2022)	
RAC875_c48025_483	4B	666204303	TraesCS4B02G389700*	WPM	2019	Winter	Adult	NA	Denmark & Sweden	Alemu et al. (2021)	Carbohydrate-binding protein
				WYR	2016	spring	Adult	NA	Canada	Iqbal et al. (2022)	
wsnp_Ku_c1876_3666308	6B	6BL_4389944*	*TraesCS6B02G310500*	WPM	2019	Winter	Adult	NA	Denmark & Sweden	Alemu et al. (2021)	Makorin RING-zinc-finger protein
				WYR	2018-2019	Spring	Adult	NA	Kazakhstan	Genievskaya et al. (2020)	
Excalibur_c49875_479	2D	634877378	TraesCS2D02G564300*	WPM	2019	winter	adult	NA	Denmark	Alemu et al. (2021)	Receptor-like kinase
				WYR	NA	winter	seedling	Warrior (-) FS 53/20	Germany	Shahinnia et al. (2023)	
RAC875_c31791_559	7B	704671177	TraesCS7B02G439100*	WPM	2020	winter	adult	NA	Lituania	Alemu et al. (2021)	Protein mak16
				WYR	NA	winter	seedling	Warrior (-) G 23/19	Germany	Shahinnia et al. (2023)	
RFL_Contig1338_2062	1D	11623275	TraesCS1D02G029500*	WPM	2019	winter	adult	NA	Lithuania and Estonia	Alemu et al. (2021)	ERD (Early-responsive to dehydration stress) family protein
				WYR	NA	winter	seedling	Warrior (-) FS 53/20	Germany	Shahinnia et al. (2023)	
				WYR	NA	winter	seedling	Warrior (-) FS 53/20	Germany	Shahinnia et al. (2023)	
wsnp_Ex_rep_c67098_65573819	1D	462741446	TraesCS1D02G393000*	WPM	2019	winter	Adult	NA	Denmark & Sweden	Alemu et al. (2021)	Dynamin-related protein
				WYR	NA	winter	seedling	Warrior (-) FS 53/20	Germany	Shahinnia et al. (2023)	
wsnp_Ra_c5346_9501281	6A	6AL_5784677*	TraesCS6A02G373200*	WPM	2019	Winter	Adult	NA	Estonia	Alemu et al. (2021)	AP-1 complex subunit gamma-1
				WSR	2016/2018	Spring	Adult	NA	Russia	Leonova et al. (2020a)	
Kukri_c39321_112	6B	151131531	*TraesCS6B02G149800*	WPM	2019	NA	Adult	NA	Denmark & Sweden	Alemu et al. (2021)	Kelch repeat-containing protein 1
				WLR	NA	NA	Seedling	NA	Canada	Fatima et al. (2020)	
BS00041742_51	3A	802590425	TraesCS3A02G490300*	WPM	2019	winter	adult	NA	Sweden	Alemu et al. (2021)	FMN-dependent NADPH-azoreductase
				WLR	2017	spring	adult	NA	Canada	Iqbal et al. (2022)	
wsnp_Ra_c3766_6947263	6B	151130562	*TraesCS6B02G149800*	WPM	2019	winter	adult	NA	Denmark & Sweden	Alemu et al. (2021)	Kelch repeat-containing protein 1
				WLR	NA	Spring & winter	seedling	MBDS	Canada	Fatima et al. (2020)	
wsnp_Ex_c64005_62986957	3B	743632624	TraesCS3B02G481500*	WPM	2019	winter	adult	NA	Denmark	Alemu et al. (2021)	Kelch repeat-containing F-box protein-like
				WLR	2017, 2019, 2020	spring	adult	NA	Canada	Iqbal et al. (2022)	
BS00021735_51	5B	593943053	*TraesCS5B02G418200*	WPM	2019	winter	adult	NA	Denmark & Sweden	Alemu et al. (2021)	Elongation factor 1-alpha
				WLR	2017/2019	winter	adult	NA	Canada	Fatima et al. (2020)	
wsnp_Ex_c19724_28720939	5B	478306307	*TraesCS5B01G292800*	WPM	2019	winter	adult	NA	Denmark	Alemu et al. (2021)	WD40 repeat-like protein
				WLR	2013- 2014	NA	seedling	BBBQD	USA	Lhamo et al. (2023)	
wsnp_Ex_c6548_11355524	5B	439725143	*TraesCS5B02G257300*	WPM	2019	winter	adult	NA	Denmark & Sweden	Alemu et al. (2021)	WRKY transcription factor
				WLR	2017-2019	winter	adult	NA	Canada	Fatima et al. (2020)	
BS00063208_51	7B	637618402	TraesCS7B02G371600*	WPM	2019	winter	adult	NA	Denmark & Sweden	Alemu et al. (2021)	F-box protein
				WLR	NA	Spring/winter	seedling	MGBJ	Canada	Fatima et al. (2020)	
RAC875_c65573_410	3A	742768234	*TraesCS3A02G528900*	WPM	2021	spring	seedling	NA	Egypt	Mourad et al. (2023)	Vacuolar-processing enzyme
				WLR	2015, 2016 &2017	NA	adult	NA	China	Zhang et al. (2021)	
Kukri_s117946_404	3A	739734944	TraesCS3A02G526000*	WPM	2019	winter	adult	NA	Sweden	Alemu et al. (2021)	Protein FLX-like 3
				WLR	2017-2019	spring	adult	NA	Canada	Fatima et al. (2020)	

Nine markers were found to be common between WPM and WYR that were located on six different chromosomes (**Figures 7A, B**). All these markers were associated with the resistance of the two diseases in two different countries except IWB10765 that was associated with the resistance of the two diseases in the same country “Australia” (**Table 5**). Moreover, all these markers were found to associated with the resistance of both diseases in adult growth stage, except markers “Excalibur_c49875_479”, “RAC875_c31791_559”, “RFL_Contig1338_2062”, and “wsnp_Ex_rep_c67098_65573819” that were associated with the resistance of WYR in seedling growth stage and WPM in adult growth stage, respectively. Remarkably, most of these identified common markers were associated with the resistance of both diseases in Europe except “IWB74”, “RAC875_c48025_483”, and “wsnp_Ku_c1876_3666308” markers that were associated with WPM resistance in Australia, Denmark and Sweden, respectively and WYR resistance in China, Canada, and Kazakhstan, respectively. Interestingly, none of the identified gene models had a functional annotation associated directly with disease resistance. However, most of them have indirect relation with disease resistance in wheat such as Dynamin-related protein controlled by *TraesCS1D02G393000* gene that was reported to interact with *TaYRG1.6* gene and produce high level of WYR resistance in wheat (Zhang et al., 2022).

Only one marker was found to be commonly associated with the resistance of WPM and WSR (**Table 5**). This marker is located on 6A and within *TraesCS6A02G373200* gene model. It was significantly associated with adult plant resistance of both diseases in different countries (Estonia and Russia) and different types of wheat (spring and winter). The functional annotation of this gene was found to control AP-1 complex subunit gamma-1. Unfortunately, little is known about the role of this protein in the resistance of different diseases in wheat. However, as this gene was associated with the resistance of two different diseases under the environmental conditions of two countries far from each other (Russia and Estonia), more concern should be given to understand its role in the resistance.

Ten markers were commonly associated with the resistance of WPM and WLR. These markers were distributed among five different chromosomes with three markers on 5B and 3A, two markers on 6B, and one marker on 3B and 7B each. These markers were located within ten different gene models (**Figures 7A, B**). Notably, the three gene models on 3A are located on the long arm of the chromosome and very near each other especially for *TraesCS3A02G528900* and *TraesCS3A02G526000*. These two gene models were associated with WPM resistance in Egypt and Sweden and WLR in China and Canda (**Table 5**). The functional annotation of these genes was found to control Vacuolar-processing enzyme and Protein FLX-like 3, respectively (**Table 5**). Vacuolar-processing enzyme was reported to play an important role in plant cell death and autophagy that occur when the plant exposed to stress (Wleklik and Borek, 2023). Cell death is a known technique of plant that innate immune responses and produce hypersensitive response (HR) hence a high level of resistance against foliar diseases (Coll et al., 2011). Little is known about the role of Protein FLX-like 3 in wheat. However, as *TraesCS3A02G526000* gene that controlling the production of this protein was significantly associated with the resistance of two different diseases in two different countries, Sweden and Canada, therefore, it is worth understanding the role of this protein in the resistance. The remaining eight markers were associated with WLR under conditions in Canada and USA and WPM in Sweden and Denmark, respectively; therefore, they could be specific for almost similar environmental conditions.

Several markers were found to be common between two rust diseases (**Supplementary Tables S10–S12**). However, we will focus on the common markers among the three rust diseases. Only two markers were identified (**Table 6**). These two markers were located on two different chromosomes, 3B and 6D, and located within two different gene models (**Figure 7**). Notably, each of these two markers was associated with the three rusts under the same geographical conditions but no marker was found to control the three rusts in different geographical regions. AX-94883935 marker located within *TraesCS3B02G592000* gene model was found to be significantly associated with the three rusts under Indian conditions at seedling and adult growth stages. This gene was found to be functionally annotated control 2-oxoglutarate (2OG) and Fe(II)-dependent oxygenase superfamily protein, one of the largest protein family in plants. It mainly contains four families which are; flavonol synthase (FLS), flavonone 3β-hydroxylase (F3H), anthocyanidin synthase (ANS)/leucoanthocyanidin dioxygenase (LDOX), and flavones synthase I (FNS I) that control important oxidation reactions (Wang et al., 2021a). The role of this protein family in disease resistance in wheat was not identified yet. However, it was reported to be involved in various vital metabolic pathways of plants (Wei et al., 2021). Phenotypic variation explained by this marker (R^2^) ranged from 2.53 to 6.60% for WSR and WYR respectively which suggested that it has a minor effect in controlling these diseases (**Supplementary Tables S1–S3**). The other marker (wsnp_Ex_rep_c68175_66950387) located within *TraesCS6D02G032300* gene was associated with seedling and adult resistance in wheat under the conditions of Kazakhstan (**Table 6**). This gene was functionally annotated to control protein kinase production in wheat. Protein kinase plays crucial roles in plant growth, immunity and development (Tang et al., 2017). The phenotypic variation explained by this marker suggested that it has a minor effect on WSR (R^2^ = 7.0%, **Supplementary Table S3**), major effects on WYR (R^2^ = 10.00%, **Supplementary Table S1**), major effect on WLR in one study [R^2^ = 13%, **Supplementary Table S2** (Genievskaya et al., 2022)] and different minor and major effects in another study [R^2^ ranged from 7.84 to 11.97%, **Supplementary Table S2** (Zatybekov et al., 2022)].

**Table 6:** List of significant markers associated with the three rusts disease [yellow rust (WYR), leaf rust (WLR), and stem rust (WSR)], their chromosomal position (bp), year of evaluation, region of evaluation, races that they resist, and gene models they are located within.

Marker	Chr	Position (bp)	Gene models	Foliar disease	Evaluation year	Wheat type	Growth stage	Races	Regions	Citation	Functional annotation
AX-94883935	3B	816277499	*TraesCS3B02G592000*	WLR	2017-2018	Spring	Seedling & adult	LR_104-2, LR_12-5, LR_77-1, LR_77-5, LR_77-9, 77-5(121R63-1), and 77-9(121R60-1)	India	Kumar et al. (2020); Vikas et al. (2022)	2-oxoglutarate (2OG) and Fe(II)-dependent oxygenase superfamily protein
				WYR	2018	NA	Seedling	110S119	India	Pradhan et al. (2020)	
				WSR	2018/2019	Spring	Seedling & adult	SR_21A2 and SR_11	India	Kumar et al. (2020)	
wsnp_Ex_rep_c68175_66950387	6D	13600765	*TraesCS6D02G032300*	WLR	2019	Spring	Seedling & adult	TQTMQ, TQKHT, and TRTHT	Kazakhstan	Genievskaya et al. (2020); Zatybekov et al. (2022)	Protein kinase
				WYR	2019	Spring	Adult	NA	Kazakhstan	Genievskaya et al. (2020)	
				WSR	2019	NA	Seedling	TKRTF, PKCTC, RKRTF	Kazakhstan	Zatybekov et al. (2022)	

**Figure 8:**
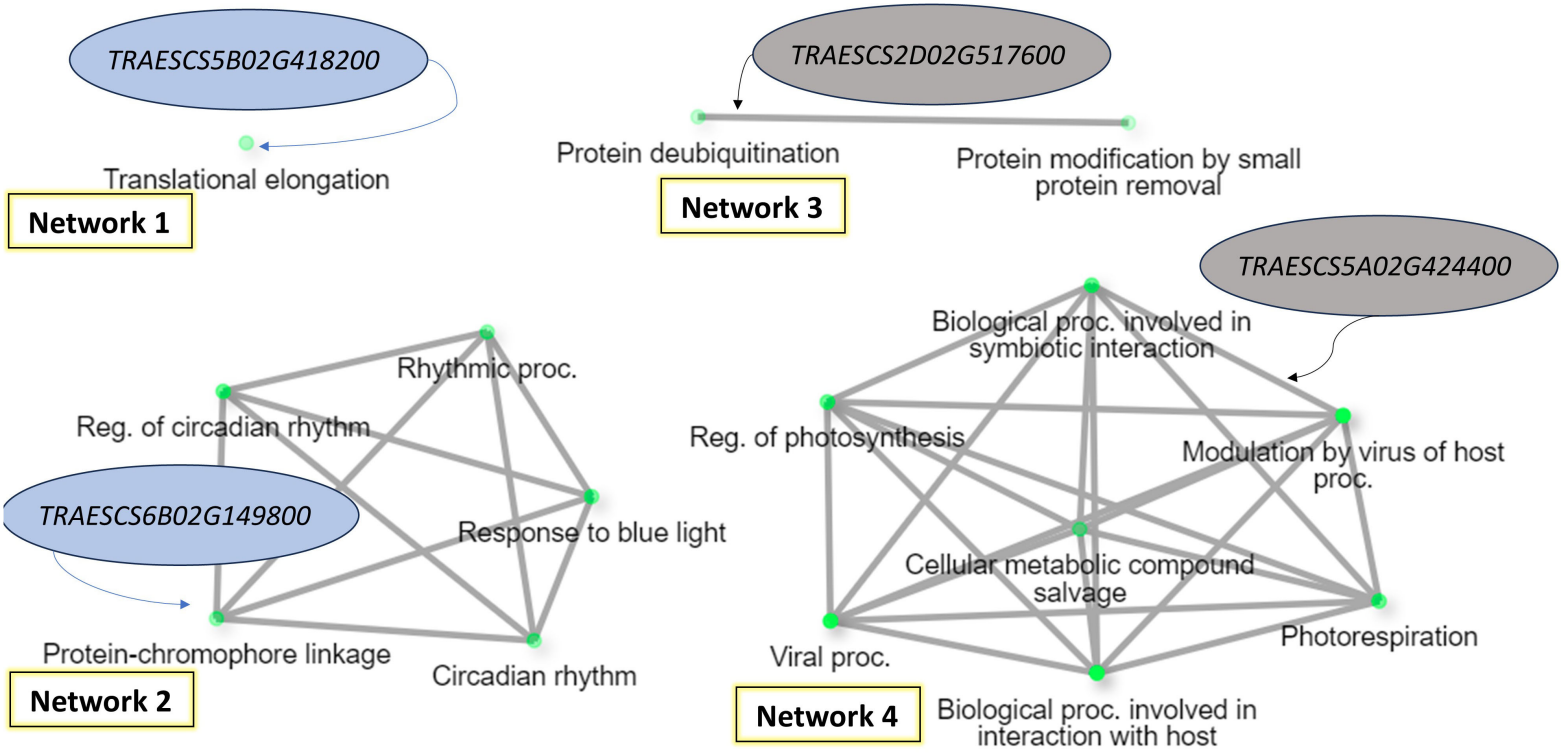
Gene enrichment of the identified gene models associated with WPM and at least one of rust disease based on the biological process (FDR = 1%).

The gene enrichment of the common gene models presented in **Tables 5**, **6** were investigated to detect if there any common biological process could control the resistance of all the four foliar diseases (**Figure 8**). Based on gene enrichment, four biological processes networks controlled by four gene models were identified (FDR<1%). Two of these networks were associated with the resistance of WPM and WLR (Network 1 and Network 2). The other two networks were associated with the resistance of WPM and WYR (Network 3 and Network 4). Interestingly, none of the identified biological processes was directly controlling fungal disease resistance in wheat. Network 1 was found to control translocation elongation, a process that allows the advances of the mRNA–tRNA moiety on the ribosome thus allowing the movement of next codon into the decoding center (Frank et al., 2007). Network 2 was found to control wheat circadian, a biological process that enables the plant to adapt to daily environmental changes and control the time to produce and consume energy (Kim et al., 2017). Network 3 was found to control protein deubiquitination, a process that affects plant development by affecting a wide range of processes, hormone signaling, including embryogenesis, and senescence (Moon et al., 2004). The last network was found to control many biological process pathways involved in interaction with host, symbiotic interaction, and modulation of host by virus. This network was involved in both WPM and WYR resistance. The biological processes of this network were associated with disease resistance/susceptibility. For example, modulation of plant immunity by virus is an essential step for disease development. Moreover, it includes some changes in plant physiology and disturbance of unrelated endogenous processes (Conti et al., 2017). As most of the identified networks were not associated directly with disease resistance, more studies should be done to understand their role in wheat resistance against fungal diseases.

In summary, due to the low number of common markers and common genes among the four reviewed fungal diseases in this article, they seem to have different genetic systems that control their resistance in wheat. Furthermore, for a single disease, low number of common markers/genes were found to commonly associated with the resistance around the world thus reflecting the complexity of their pathogen, regional germplasm use, and the urgent need for investigating more studies to obtain broad-spectrum resistance. However, the identified common markers/genes in this review could be a good start to achieve this goal. Surely, more studies and investigation should be done to understand their role in the resistance. It is highly recommended also that the expression of common genes (among diseases or among countries) should be tested and verified across years or/and locations. Then, specific primers (KASP or traditional primers) can be designed and used in screening genotypes to broad-spectrum resistance to foliar diseases in wheat.

## 6 Conclusion

Wheat rusts (yellow, leaf, and stem rust) and powdery mildew are important foliar diseases affecting wheat yield around the world. Many efforts have been done to improve wheat resistance to these diseases that lead to new phenotyping methods and identifying genes controlling the resistance. In this review, some genes were found to be commonly associated with resistance in different regions around the world. These genes are very important in producing high levels of broad-spectrum resistance. Furthermore, common genes controlling the resistance of different diseases were investigated. However, very low number of common genes were found that confirming the presence of different genetic systems controlling the resistance of each disease. Therefore, producing genotypes that are resistant to different diseases is very complex.
